# Percutaneous placement of the peripheral catheter to the subclavian vein for a VA shunt

**DOI:** 10.11604/pamj.2017.27.42.11374

**Published:** 2017-05-16

**Authors:** Drosos Evangelos, Giakoumettis Dimitrios, Sfakianos Georgios, Eleftherakis Nikolaos, Papadopoulos Filippos, Themistocleous Marios

**Affiliations:** 1 Department of Neurosurgery, University of Athens Medical School, “Evangelismos” General Hospital, Athens, Greece; 2 Department of Neurosurgery, Children’s Hospital “Agia Sofia”, Athens, Greece; 3 Department of Cardiology, Children’s Hospital “Agia Sofia”, Athens, Greece

**Keywords:** Ventriculo-atrial shunt, hydrocephalus, percutaneous, subclavian vein

## Abstract

Hydrocephalus is a common neurosurgical pathology that affects people of all ages and especially the pediatric population. It can be very often a life threatening condition that pediatric neurosurgeons must deal with. Therefore a number of CSF diversion techniques have been established. The gold standard treatment currently is the placement of a ventriculo-peritoneal shunt. Because of hydrocephalus being a lifelong condition, it is almost in daily practice dealing with cases of shunt failures for a number of reasons. Herewith we present a 4 year old child with multiple ventriculo-peritoneal shunt revision surgeries and ventriculo-atrial failure due to distal catheter malfunction that was treated with percutaneous placement of the peripheral catheter in the subclavian vein.

## Introduction

Hydrocephalus is one of the most common neurosurgical pathologies with an estimated prevalence of 1-1.5% in the general population Recent epidemiologic data in children suggest a prevalence of 1.1/1000 whereas infants with congenital hydrocephalus are presented in 0.9-1.8/1000 births . It is a potentially life threatening condition and therefore a number of procedures have been suggested over the years. The main goal has always been cerebrospinal fluid (CSF) flow diversion, which is the most common procedure in pediatric Neurosurgery. Currently the use of ventriculo-peritoneal (VP) shunts is considered the gold standard followed by implantation of the peripheral catheter in the right atrium through the jugular vein. Other implantation sites have also been used during the years; however, they were abandoned or used as second-line solutions due to the high rate of complications they presented [1].

## Patient and observation

A 4-year-old boy presented in the emergency department with hydrocephalus due to ventriculo-peritoneal shunt malfunction and because of no alternatives he was treated with a ventriculo-atrial (VA) shunt which was percutaneously inserted through the subclavian vein. The child had been diagnosed at the age of two months with an intramedullary spinal cord tumor with pathology of primitive neuroectodermal tumor (PNET) grade IV according to World Health Organization. At first he presented with left hemiparesis and underwent brain and spinal MRI that revealed hydrocephalus and a 4.7 × 1.5 cm intramedullary lesion extending from the second cervical to the first thoracic vertebrae. Furthermore there was a leptomeningeal spread not only intracranially but in the spinal canal as well. The patient underwent two operation procedures. Firstly, a ventriculo-peritoneal shunt (Medtronic PS Medical) was placed to deal with hydrocephalus and then a radical resection of the tumor was performed; nevertheless, some small tumor remnants were left behind because they could not be safely removed. The postoperative period was complicated with prolonged stay in the pediatric intensive care unit due to postoperative neurological deterioration. During his stay in the ICU the patient experienced an episode of cardiac arrest and a permanent internal defibrillator was implanted through the right subclavian vein. Finally he was discharged from the neurosurgical department with severe left hemiparesis and he was further treated with high-dose chemotherapy. In the following years he underwent four revision surgeries, which included several revisions of the distal part and the introduction of a new proximal catheter at the opposite lateral ventricle, due to VP malfunction caused by peritoneal malabsorption.

**Figure 1:**
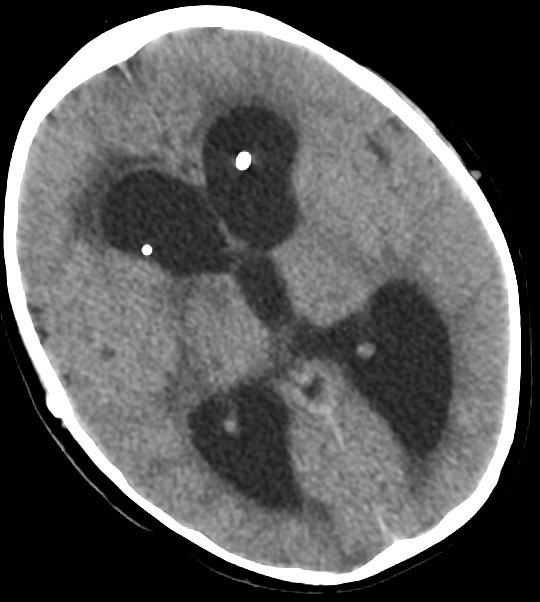
Preoperative CT scan demonstrating hydrocephalus

**Figure 2:**
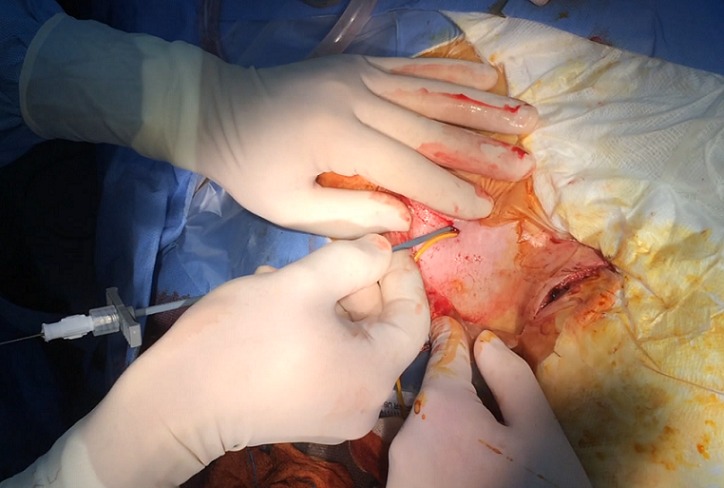
Insertion of the peel-away sheath through the guide-wire

**Figure 3:**
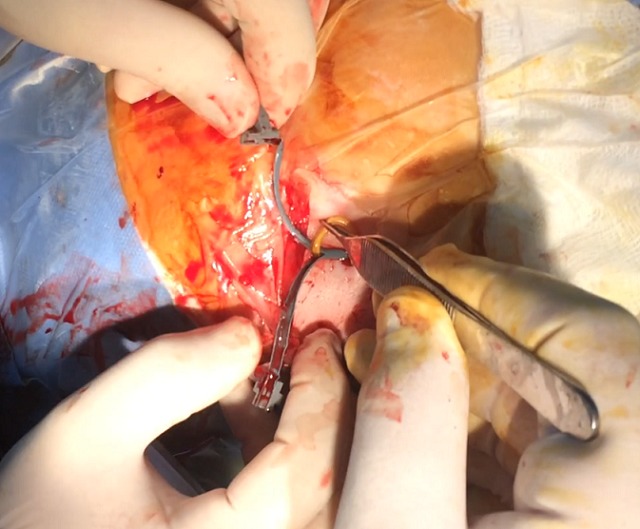
Peripheral catheter inserted through splitting dilator

**Figure 4:**
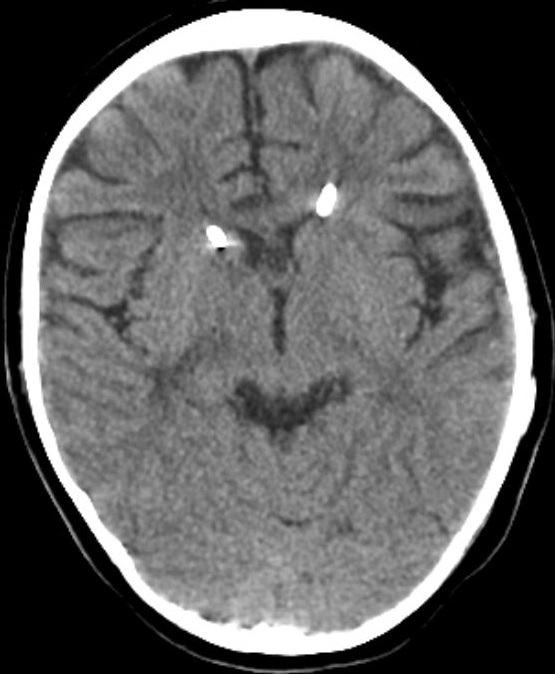
One year follow up CT scan showing the Cather sin place and the normal ventricular system

In the last operation, the distal catheter was implanted to the right internal jugular vein. At the age of 4 years old the child presented to the emergency department with deteriorating level of consciousness and vomiting. The shunt was tapped and demonstrated increased pressure with good proximal flow and absence of distal runoff. A computer tomography (CT) scan revealed ventricles dilatation and periventricular edema Figure 1. Moreover an ultrasound imaging of the neck veins revealed bilateral internal jugular vein thrombosis. Due to lack of alternative solutions, a decision was made to implant the distal catheter in the right atrium through the left subclavian vein with the help of the interventional cardiologist of the hospital. The peripheral catheter was introduced percutaneously using the Seldinger technique. Tunneling was done up to the clavicle region and a stab wound was performed at the puncture site. The subclavian vein was punctured using a 21 G needle and a 0,018″ guide wire was introduced. Through that, a 4-French sheath was introduced and the wire was withdrawn. Afterwards, a new guide wire 0,035″ was introduced and the sheath was also withdrawn. In order to achieve gradual vein dilation and to avoid injury of the vessel two consecutive sheaths of 6 and 7 French accordingly were used. The latter was a peel-away sheath Figure 2. The wire and the introducer were withdrawn and the distal catheter was introduced through the peel away dilator Figure 3. Placement of the catheter in the right atrium was confirmed with an intra-operative x-ray with the C-arm. The dilator was peeled away and the wound was closed in a standard manner. The patient made an uneventful recovery with no post-operative complications and he was discharged free of symptoms of increased intracranial pressure a few days later. At one year follow-up the child is without symptoms of hydrocephalus and the CT scan shows a normal ventricular system with both catheters in place Figure 4.

## Discussion

Ventriculo-peritoneal shunt malfunction is a common problem that Neurosurgical departments, especially in Pediatric institutions, come across. There are times that a patient needs multiple revisions for a shunt malfunction and therefore there is a need for reliable alternatives. Numerous locations of planting the distal part had been used in the past in cases that the routine locations were no longer feasible. The list includes pleural cavity, gallbladder, urinary bladder and stomach. Ventriculogastrostomy has been described in the past in dogs as well as in humans but has never been used systematically due to catheter contamination and increased risk of infection [2]. Gallbladder, however, offers an environment where CSF water and electrolytes can be effectively absorbed without being affected from the pressure changes during bladder contractions or predisposing to gall stones formation with the most common complication reported being shunt infection (reaching up to 32% of reported cases). Moreover, bile reflux and chemical ventriculitis have been reported with fatal consequences in one case [3]. Finally, even without the catheter being the cause, synchronous gallstones presence can cause catheter obstruction and shunt malfunction [4]. On the other hand, urinary bladder or ureter is low pressure cavities that can be utilized. They form a fairly sterile environment with constant flow reducing the risk for obstruction. Nevertheless, obstruction from urinary track stones has been reported [5]. Moreover the constant flow can cause fluid and electrolyte disturbances due to excess losses. A reliable second line target for CSF drainage is the pleural cavity. When combined with good patient selection and antisiphoning valves it can be effective in a long term basis in both pediatric and adult populations [6]. Complications related to this method include pleural effusion and subsequent hydrothorax especially in patients under 7 years old, pseudocyst formation and pleural empyema [6]. The most commonly used second line method is the ventriculo-atrial shunt [1]. A number of routes to the right atrium have been described. Usually, the facial vein or the external jugular vein are dissected and isolated and used as an entrance site to the venous system. This method is usually assisted either by endovascular techniques [7], venography in cases of complex anatomy [8] or by transesophageal ultrasound [9]. Other entrance sites such as femoral vein [10] or transverse sinus [11] have also been reported.

The distal catheter can be introduced to the right atrium through the azygos vein [12] or even directly through the pericardium[13]. Both methods require a thoracotomy to be performed. This is a procedure that should be performed with the assistance of a thoracic surgeon. However, even in this case, the complications from the airway (edema, injury, gastric content aspiration), the lungs (atelectasis, bronchospasm, pneumo - hemo- chylothorax, fistula formation), the heart (hematoma, pain, arrhythmia, pulmonary hypertension) or the chest wall (nerve damage, post-thoracotomy pain syndrome) can be proved life-threatening [14]. Even though with these methods the vessels of the neck are spared and can be used for long term treatment (e.g. parenteral nutrition) such procedures are rarely used [12]. With hydrocephalus being an emergency requiring immediate treatment, especially due to shunt malfunction, employing such a method is very important. Subclavian vein catheterization is a technique most surgeons are familiar with. Thus the learning curve is smaller than that of peritoneal cavity opening. Furthermore, it limits the need for assistance from a surgeon from a different specialty, contributing in the simplicity of the procedure. Moreover, due to the location of the vessel being under an anatomically stable landmark such as the clavicle, it can be performed in both adults and children, even in obese patients, as opposed to peritoneum or neck veins dissection. Another important advantage of this method is the limited use of the jugular veins. Endovascular placement of the peripheral catheter of a shunt has been reported to lead in thrombosis of the vessel and subsequent shunt malfunction [15]. That way in cases like the one we described where both veins were occluded, a safe and accessible route to the right atrium is provided. Even though hybrid techniques with endovascular recanalization of the jugular vein have been documented [16], they require the presence of specialized personnel and equipment that are not always available in many parts of the world.

Regarding the safety of the method, the use of consecutive sheaths of increasing diameter is an important point. Vessel injury can be the cause of a hematoma that may require aspiration [17]. At the same time endothelial injury is considered the cause for vessel thrombosis and subsequent shunt malfunction [17]. Therefore, it is safer practice to dilate the vessel in a more gradual way leading to fewer chances of such complications. Another interesting fact is that both these complications are reported to occur more commonly with internal jugular vein catheterization than with subclavian vein, thus raising the issue of using the subclavian vein as an entrance point before considering the jugular vein as a matter for discussion for future reports [17]. Another important factor of our method, which is an important factor for the worldwide medical community, is the cost of the operation. Decreased operative time and site exposure result in relatively less infections therefore a decreased overall cost. Further cost reduction is offered by the use of the relatively cheaper sheaths and guides that are used in interventional cardiology as was previously described. Nevertheless, the use of these types of vessels bares drawbacks. The most common intraoperative complication described is the accidental artery or lung puncture and subsequent hemo/pneumothorax [17]. A case of accidental endotracheal tube cuff puncture has also been described [18]. Concern can also raise the need for synchronous parenteral nutrition administration which is considered a risk factor for bacteremia and fungemia. However, there have been no consistent data proving or disproving these concerns [19]. Serious long term complications have been reported, relating mostly with latent shunt infection and misplacement of the tip of the catheter [20]. Cardiac tamponade due to pericardial effusion and recurrent pulmonary embolism with subsequent pulmonary hypertension and cor pulmonale as well as cardiac thrombus formation [20] can be the cause for shunt malfunction or even proved life threatening. Last but not least in pediatric populations eventual migration of the catheter due to child growth increases the difficulty for the revision of the distal catheter.

## Conclusion

The goal of surgery besides providing treatment, has always been presenting new techniques based on three principles: effectiveness, safety and convenience. Here we presented the percutaneous insertion of the distal catheter of a VA shunt in the right atrium through the subclavian vein and at the same time protection of the vein with careful and gradual dilation. It is a method that can be effective for complex cases, safely done and easy and quick to perform.

## Competing interests

The author declare no competing interests.
